# Sound Sensitivity of the Saccule for Low Frequencies in Healthy Adults

**DOI:** 10.1155/2013/429680

**Published:** 2013-10-24

**Authors:** Seyede Faranak Emami, Akram Pourbakht, Ahmad Daneshi, Kianoush Sheykholeslami, Hessamedin Emamjome, Mohammad Kamali

**Affiliations:** ^1^Department of Audiology, School of Rehabilitation, Iran University of Medical Sciences, Mirdamad Street, 98, Tehran 1545913187, Iran; ^2^Department of Audiology, Rehabilitation Research Center, School of Rehabilitation Sciences, Iran University of Medical Sciences, Tehran, Iran; ^3^ENT-Head and Neck Research Center, Hazrat Rasoul Akram Hospital, Iran University of Medical Sciences, Tehran, Iran; ^4^Department of Surgery, University of Illinois, Rockford, USA; ^5^Audiology Research Center, Hazrat Rasoul Akram Hospital, Iran University of Medical Sciences, Tehran, Iran; ^6^Rehabilitation Research Center, School of Rehabilitation Sciences, Iran Univessity of Medical Sciences, Tehran, Iran

## Abstract

Approximately 80 years ago John Tait speculated about a possible auditory role for the otolith organs in humans those days, there was no direct evidence for that idea. This time is for us to review and research. Then, the objective of our study was to investigate saccular hearing in healthy adults. We selected twenty healthy controls and twenty-four dizzy cases. Assessment comprised of audiologic evaluations, cervical vestibular evoked myogenic potentials (cVEMPs), and recognition of spoken phonemes in white noise (Rsp in wn). In the case group (a total of 48 ears), the cVEMPs abnormalities were all unilateral (24 affected ears and 24 contralateral unaffected ears). Affected ears with decreased vestibular excitability as detected by abnormal cVEMPs had decreased Rsp in wn (mean = 60.78 ± 8.33), whereas both unaffected (mean = 96.24 ± 2.4) and control ears (mean = 96.24 ± 2.4) presented normal results. The correlation between RSP in wn and p13 latencies was significant (*P* < 0.05, *r* = −0.551). The peak-to-peak amplitudes showed significant correlation to RSP in wn (*P* < 0.05, *r* = 0.307). The correlation between RSP in wn and the latencies of n23 was significant (*P* < 0.05, *r* = −0.493). We concluded in presence of severe competing noise, saccule has a facilitating role for cochlea and can improve to detection of loud low-frequencies.

## 1. Introduction 

There are afferent fibers in the vestibular nerve of amniotes (reptiles, birds, and mammals) that respond to sound at levels within the normal range of hearing. The ascending auditory pathway (inner ear, cochlear nucleus, medulla, midbrain, thalamus, and cerebrum) of amniotes is organized similarly to those of anamniotes (fish and amphibians). Convergent neuroanatomical specializations in two species may reflect common functional requirements [1, 2]. These structures have retained sound sensitivity in man and primates [3, 4].

Intense air-conducted stimulations with low frequencies (between 50 and 800 Hz) may evoke the continuous responses in the human saccular neurons. The range of acoustic sensitivity of the sacculus happens to coincide with the range of voice pitch, for male voices between 80 and 200 Hz and up to 400 Hz for females. Also, the first formant of our voice falls within the range of saccular sensitivity [4–6]. Thus, given the proximity of the ear to the larynx, it is possible that saccular responses may be obtained to an individual's own vocalisations, particularly for singing. Another possibility is that responses are obtained when there are large groups of individuals vocalising together, such as in a choir or a crowd at a concert or sporting event [6–8].

On the other hand, most acoustically responsive fibers in the inferior vestibular nerve have irregular spontaneous activity and originate in the saccule. These fibers traced centrally arborized extensively in vestibular nuclei and ventromedial to the cochlear nucleus. Then, mammalian saccule responds to sound and sends acoustic information to the central nervous system [2–9], including bulbospinal, brain-stem, cerebellum, higher centres up to the hypothalamus [2, 6, 7], and medial and superior temporal gyrus [10]. Therefore, the objective of this study was to investigate saccular hearing in healthy adults.

## 2. Materials and Methods

### 2.1. Participants

This case-control study consisted of twenty healthy controls (11 females, 9 males) and twenty-four dizzy patients (14 females and 10 males), which were screened from of sixty-seven patients with benign paroxysmal positional vertigo and migraineurs. They were presented to the Audiology Department of Hazrat Rasoul Akram hospital of IRAN university of medical sciences (Tehran, Iran), from May 2012 to December 2012. We screened all volunteer eligible patients about seven months (we could not find previous collected works and used of censes method for sampling strategy). 

The diagnose of patients with benign paroxysmal positional vertigo was based on medical history and findings of characteristic nystagmus (torsional up beating nystagmus with latency and fatigue lasting less than 1 min) and subjective vertigo in the Dix-Hallpike test [11, 12].

In migraineurs vestibular dysfunctions were connected with nystagmus, and with episodic vertigo, or a variety of combinations of headache/vertigo. A number of patients reported that their symptoms were worse with achieved head status, but this is not confused with BPPV, since patients with migrainous vertigo were nauseated or phonophobic during attacks [11, 12].

The *exclusion criteria *consisted of history of ear infections and middle ear diseases, which can interfere with cVEMPs measurements, and conditions that can cause abnormal auditory function. To this list were included history of head trauma, ototoxic drugs, otosclerosis, labyrinthitis, cardiac and metabolic diseases, heart failure, anemia, hypothyroidism, hyperthyroidism, diabetes mellitus, hypertension, and various neurological diseases (vertebrobasilar insufficiency, temporal lobe epilepsy, multiple sclerosis, central nervous system tumors, and cerebellar infarction, among others).

The inclusion criteria involved normal function of hearing, middle ear pressure, olivocochlear and auditory brainstem pathway, with abnormal function of saccule. 

A handedness questionnaire was also administered. All the subjects were right-handed they were native speakers of the Persian language (with unilinguistic abilities). They received detailed information about the study and the testing that would be involved. All of the tests performed on the same day and in each step of evaluation when the procedure was completed for the one test, subjects were given a short break and the whole procedure repeated for another. The study was approved by the IRAN university ethics committee.

### 2.2. Ethical Considerations

Our study was on human subjects, so to minimize harms and risks and maximize benefits and respect human dignity, privacy, autonomy, immunity, safety, respectability, and satisfaction, we took human precautions with our case-control groups and strived to distribute the benefits and burdens of research fairly. In addition, our research did not present the work of others as their own or did not fail to give appropriate credit for the work of others through citations.

### 2.3. Assessments

Evaluation for screening of case group, consisted of *pure tone audiometry, tympanometry and acoustic reflex test, auditory brainstem response, electronystagmography, word recognition scores (Rsp) in quiet, and cervical vestibular evoked myogenic potentials (cVEMPs),* which were employed for reviewing our inclusion criteria. Recognition of spoken phonemes in white noise (Rsp in wn) and cVEMPs involved assessment of our main variables. 

The devices were diagnostic pure tone audiometry (Madsen:OB-822), impedance acoustic metr (Maico MI 34), full system of auditory-vestibular evoked potentials (Labat Epic-plus).

Pure tone thresholds in the normal range (−10 to 25 dBHL) were obtained from each person's over the frequency range of 250–8000 Hz [13]. Tympanometry served to evaluate the middle ear status [14]. Acoustic reflex test (Ipsilateral and contralateral) was conducted to eliminate the possibility of any additional auditory olivocochlear pathology. The normal range considered from about 85 to 100 dB SPL for pure tone stimuli [15]. *Auditory brainstem response* was done to exclude the probability of any additional brainstem pathology. We considered the responses to be abnormal when peaks III and/or V were absent or when the peak to peak I–V exceeded the normal limits of our laboratory (4.40 ms for females, 4.58 ms for males). Electronystagmography used to reject the possibility of any additional vestibular pathology. The battery of electronystagmography tests included assessment of the central vestibular and vestibulocular systems with evaluation of gaze [12]. Word recognition scores in quiet presented by one female (monitoring of live voice). The normal scores were 96–100% [16].

#### 2.3.1. Cervical Vestibular Evoked Myogenic Potentials (cVEMPs)

During the cVEMPs, recording patients were instructed to turn and hold their heads as far as possible toward the side contralateral to the stimulated ear. At that point, the overall electromyogenic activity of the sternocleidomastoid muscle was set as the reference level of the tonic contraction. Patients were asked to maintain contraction at this level throughout the test session. The active electrode was placed over the middle portion of the ipsilateral sternocleidomastoid muscle body as this location appears to generate the most reliable and consistent responses [17]. 

The reference and the ground electrodes were placed over the upper sternum and on the midline forehead, respectively [5]. Auditory stimuli consisted of tone burst (500 Hz, 120 dB peak SPL, rise/fall time = 1 ms, plateau = 2 ms), presented to the ear ipsilateral to the contracted sternocleidomastoid muscle, band pass filtered (20 Hz to 2 kHz), and a grand average of the 200 responses calculated by a standard evoked potential recorder. The latencies, amplitudes, and peak to peak amplitudes of these waves were calculated and recorded. The cVEMPs results for the control group were used as normative data. The normative values for latency and cVEMPs asymmetry ratio were calculated as mean ± two standard deviations. For each subject, the cVEMPs asymmetry ratio (evoked potential ratio) was calculated according to the formula of Murofushi et al. 100[(*A*_*n*_ − *A*_*d*_)/(*A*_*n*_ + *A*_*d*_)], where *A*_*n*_ (normal) = p13 − n23 (the peak-to-peak amplitude in the *normal ear*) and *A*_*d*_ (disease) = p13 − n23 (the peak-to-peak amplitude in the *affected ear*) [2]. 

Any cVEMPs asymmetry ratio above the calculated upper limit was considered to reflect depressed response on the side with lower amplitude findings and was interpreted as abnormal. The latencies longer than the calculated upper limit were interpreted as abnormal. Absence of a meaningful waveform with p13 and n23 (no response) was also considered as an abnormal finding [5]. 

#### 2.3.2. Recognition of Spoken Phonemes in White Noise (Rsp in wn)

Rsp in wn was used to study low-frequency sensitivity [18, 19]. Regarding phonological properties of the persian language, and since after onset of voiced consonants the frequencies of spectral peaks move toward the frequencies that define the following vowel [19]. We combined the vowel */e/, *which stimulates low-frequency neurons [20], with ten voiced consonants (/*m*/, /*n*/, /*w*/, /*gh*/, /*b/, /d/*, /*z*/, /*g*/, /r/, /l/). Then, we created two homogeneous monosyllabic phoneme consonant-vowel lists ( *List-1:* /*me*/, /*ne*/, /*we*/, /*ghe*/, /*be*/, /*de*/, /z*e*/, /*ge*/, /re/, /le/, and *List-2:* /re/, /*be*/, /*ne*/, /*we*/, /*ghe*/, /le/, /*me*/, /z*e*/, /*de*/, /*ge*/). These low-frequency phonemes with first formant lesser than 1000 H_Z_ were similar in structure and intelligibility. We assessed their signals via short-time frequency analysis, which was performed on a “segment-by-segment” basis and loaded into the Matlab workspace. They presented at 10 dB signal to noise ratio (signal = 95  dB_HL_ and white noise = 85  dB_HL_) to subjects' ipsilateral test ear, at the same time. 

The test was done by one female speaker, who was a native of the persian language and had not the dialect (there is no noticeable systematic differences in consonant scores, voicing scores, and consonant confusions for male and female talker utterances [16]). She did not know about the case or the control subjects testing was blinded and the monitoring of live voice was done. The linguistic and psychological factors of familiarity, redundancy, and emotional loading were controlled from person to person. 

### 2.4. Statistical Analysis

All analysis was done by means of the statistics software SPSS_17_. Data were expressed as mean ± standard deviation and as percentages. Kolmogorov-Smirnov test was used for evaluation of normal test distribution. One-way ANOVA was used to compare findings among the three groups. Tukey's least significant difference (Tukey HSD) test was chosen as the post hoc test. Also, Spearman's rank correlation coefficient (Spearman's rho) calculated the relationship between the groups. *P* value of <0.05 was considered to indicate statistical significance. 

## 3. Results

We evaluated twenty healthy controls (mean age 31 years and range 18–53 years), compared to twenty-four dizzy patients (mean age 33 years and range 21–54 years). All of them had normal function of hearing, middle ear, and olivocochlear-and auditory brainstem pathway.

### 3.1. Recognition of Spoken Phonemes in White Noise (Rsp in wn)

The scores of Rsp in wn obtained for the control group (mean = 96.4 ± 1.93%, minimum = 90.47%, maximum = 100%). Affected ears of the case group had decreased Rsp in wn (mean = 60.78 ± 8.33, minimum = 52.45%, maximum = 88%), (Table 1) while, unaffected ears revealed normal values (mean = 96.24 ± 2.4%, minimum = 92%, maximum = 100%).

### 3.2. Cervical Vestibular Evoked Myogenic Potentials (cVEMPs)

The mean latencies at p13 and n23 in control group (40 normal ears) were 13.37 ± 1.9 ms and 19.56 ± 2.52 ms, respectively (Table 2). The upper limits p13 and n23 latencies were 17.17 ms and 24.6 ms, respectively. The mean peak-to-peak amplitude was 47.57 ± 38.59 *µ*v, and the upper limits for this ratio were 23.46%. In the case group (a total of 48 ears), the cVEMPs abnormalities were all unilateral (24 affected ears and 24 contralateral unaffected ears). They included both decreased amplitudes and delayed latencies in nine (5 benign paroxysmal positional vertigo + 4 migraineurs) and absent responses in fifteen (13 BPPV + 2 migraineurs). 

In affected ears of benign paroxysmal positional vertigo, the mean p13 latencies and n23 latencies were 18.5 ± 1.4 ms (upper limit = 21.3 ms) and 26.95 ± 1.5 ms (upper limit = 29.95 ms), respectively. The mean peak-to-peak amplitude was 37.08 ± 11.7 *µ*v. 

In affected ears of migraineurs, the mean latencies at p13 (upper limit = 21.47 ms) and n23 (upper limit = 27.48 ms) latencies were 16.81 ± 2.33 ms and 25.08 ± 1.2 ms, respectively. The mean peak-to-peak amplitude was 33.71 ± 10.48 *µ*v. In all dizzy patients, the cVEMPs asymmetry ratio findings indicated depressed response on the side with lower amplitude findings in a single ear only.

### 3.3. The Main Outcomes

Multiple comparisons of mean p13 latencies, mean n23 latencies, and mean peak-to-peak amplitude between the three groups (affected ears, unaffected ears and control ears) were significant (*P* < 0.05 for all, one-way ANOVA test). 

Comparisons of mean p13 latencies in the affected ears versus the unaffected ears and the control group were significant (*P* < 0.05, Tukey HSD). Comparisons of mean n23 latencies in the affected ears versus the unaffected ears and the control group were significant (*P* < 0.05, Tukey HSD). Comparisons of mean peak-to-peak amplitude in the affected ears versus the unaffected ears and the control group were significant (*P* < 0.05, Tukey HSD). 

The investigation of the relationships between cVEMPs values (mean p13 latencies, mean n23 latencies, and mean peak-to-peak amplitude) and RSP in wn scores were done via Spearman's rho, because the distribution of RSP in wn was not normal. The correlation between RSP in wn and p13 latencies was significant (*P* < 0.05, *r* = −0.551, *n* = 73). The peak-to-peak amplitudes showed significant correlation with RSP in wn (*P* < 0.05, *r* = 0.307, *n* = 73). The correlation between RSP in wn and the latencies of n23 was significant (*P* < 0.05, *r* = −0.493, *n* = 73). We obtained a relationship between cVEMPs values and Rsp in wn scores.

## 4. Discussion

In this study, we found that the affected ears with decreased vestibular excitability as detected by abnormal cVEMPs had decreased Rsp in wn whereas both unaffected and control ears presented normal results. cVEMPs in response to 500-Hz tone bursts is a vestibular origin test, which can evoke by loud low-frequency sound [6] and Rsp in wn has cochlear source with similar conditions for stimulation [21], we concluded with the fact that there is a connect between them. Ring of their connection may be the presence of a low-frequency high-intensity tone, which can stimulate both saccular and cochlear afferents. Then, saccular stimulation to sound has a compensatory role for cochlear hearing in noisy conditions. The saccule has an effective sensitivity and can cooperate to detect on aloud low frequencies. Consequently, the affected ears of our study with abnormal saccular function show slight auditory abnormalities in hard of hearing situation.

Indeed, the auditory brain is responsible for sound (including speech) identification and localization. The multiple cortical and subcortical areas were involved in hearing and listening, which occupy the temporal lobe, the frontal and parietal lobes, the brainstem, and the limbic system [22]. Also, some areas of the human auditory brain (the precuneus, the precentral gyrus, the medial temporal gyrus, and the superior temporal gyrus) can be activated by saccular stimulation. These brain regions are activated in response to stimuli that can be used clinically to evoke the cVEMPs [10]. Therefore, saccular stimulation by sound can induce a possible sensation to improve better hearing in clamor locations. 

We strictly belive that, during auditory function, the range of saccular sensitivity to low frequency cues is very important. Because the neurons at the brainstem and primary auditory cortex are responsive to the low-frequencies [22] human saccule is activated by low-frequency sounds and sends effective acoustic information to the central auditory system. It should allow for sufficient speech coding for intelligibility assums connectivity to the auditory neuraxis [23].

The human saccular resonance is about 350 Hz [2, 7], while the modulation frequencies relevant to human perception span a range from 1 to 1000 Hz. In fact, low temporal modulation frequencies dominate communication sounds in many species. For human listeners, from 30 to 300 Hz a sensation referred to as roughness. In speech this range is associated with the occurrence rate of syllables and phonemes, and in music it covers faster rhythms and sequences of notes [24]. This range partially overlaps with that of the fundamental frequency in speech and the pitch of musical instruments [20]. 

The frequencies between 300 and 800 Hz create a percept of tonal quality or periodicity pitch [24]. Such sounds are usually heard during neural synchronization. It is likely that increased synchronization of auditory cortical neurons will similarly enhance the transmission of information to subsequent stages in auditory processing [23]. Then, low-frequency components, which can stimulate saccular afferents as important contributors in the neural phenomenons and may serve as the basis for hierarchical synchronization function through which the central nervous system processes and integrates sensory information [25].

Gain of the saccular system appears to be higher than the cochlear system, which would explain the compulsion to exposure to loud sounds [8]. The cochlear affective response peaking at about 90 dB SPL [26] and the saccular affective response peaking at about 130 dB SPL [8]. Indeed, auditory sensitivity of the saccule can contribute to intensity discrimination of natural sounds (voice intonation in speech, singing, crowd actions,and percussive music) [7, 23, 27]. 

The recent experience demonstrates the phonetic role of saccule in the regulation of the human voice and provides the basis for further development of this topic. The high response of the saccule allows phonemic self-regulation, compensating the low/absent tone-verbal feedback [27]. After all, we conclude in hard of hearing conditions, saccule has a facilitating role for cochlea and can contribute to the detection of high-intensity low-frequency tone. 

### 4.1. Implications for Clinical Practice

 We recommend that the cVEMPs evaluation should be done in the battery approach tests of the auditory function for normal populations. It can be a sign of the changes that are taking place in low-frequency detection abilities.

## Figures and Tables

**Table 1:** The mean of recognition of spoken phonemes in white noise in healthy controls and dizzy cases.

Subject	Healthy ears	Unaffected ears	Affected ears
Mean	96.4% ± 1.93	96.24% ± 2.4	60.78% ± 8.33

**Table 2:** The mean of latencies at p13 and n23 and peak-to-peak amplitude in healthy controls and affected ears of the dizzy cases.

Subject	P13 latency (ms)	N23 latency (ms)	Amplitude (*μ*v)
Affected ears of the patients with benign Paroxysmal positional vertigo	18.50 ± 1.40	26.95 ± 1.50	37.08 ± 11.70
Affected ears of the migraineurs	16.81 ± 2.33	25.08 ± 1.20	33.71 ± 10.48
Healthy ears	13.37 ± 1.90	19.56 ± 2.52	47.57 ± 38.59

## Abbreviations

cVEMPs: Cervical vestibular evoked myogenic potentials
Rsp in wn: Recognition of spoken phonemes in white noise.
